# Paving the Way for Ageist Attitudes through Children’s Books

**DOI:** 10.1093/geroni/igab046.3630

**Published:** 2021-12-17

**Authors:** Lena-Emilia Schenker, Jennifer Bellingtier

**Affiliations:** Friedrich Schiller University Jena, Jena, Thuringen, Germany

## Abstract

Older adults are underrepresented and rarely appear in major roles in children’s literature. According to developmental intergroup theory, numerically smaller groups are likely to become targets of stereotypes and prejudice. Because parental ageist attitudes are related to those of their children, and parents typically choose their children’s literature, we investigated parental preferences for books featuring older and younger adults and what factors might predict this preference. In an online survey, 176 parents of children aged 12 or younger rated children’s book covers featuring a child and a prominent younger or older adult. There were two identical versions of each book cover on which only the age of the adult varied. Each respondent viewed covers featuring older and younger adults, but only saw one version of each cover (i.e., counterbalanced design). Parents indicated their preference for the books by stating how much they and their children would like the book and how likely they would be to buy it. Stereotypical expectations regarding the books’ storylines were rated on a semantic differential scale (e.g., modern vs. old-fashioned). Results revealed that there were no significant differences in preferences for books featuring younger, compared to older adults. However, a stronger difference in preference for books featuring younger, over older adults was predicted by the extent of stereotypical expectations regarding the storylines. In particular, this preference was stronger in parents who expected stories with older adults to conform to prevailing ageist stereotypes, suggesting that ageist expectations may deter some parents from books featuring older adults.

